# Non-Thyroidal Illness Syndrome in Patients Exposed to Indoor Air Dampness Microbiota Treated Successfully with Triiodothyronine

**DOI:** 10.3389/fimmu.2017.00919

**Published:** 2017-08-07

**Authors:** Taija Liisa Somppi

**Affiliations:** ^1^Amplia Clinic, Medical Center, Tampere, Finland

**Keywords:** water-damaged building, mycotoxins, hypothyroid symptoms, triiodothyronine, stress intolerance, latent adrenal insufficiency, FT3/rT3 ratio, DIO2

## Abstract

Long-term exposure to dampness microbiota induces multi-organ morbidity. One of the symptoms related to this disorder is non-thyroidal illness syndrome (NTIS). A retrospective study was carried out in nine patients with a history of mold exposure, experiencing chronic fatigue, cognitive disorder, and different kinds of hypothyroid symptoms despite provision of levothyroxine (3,5,3′,5′-tetraiodothyronine, LT4) monotherapy. Exposure to volatile organic compounds present in water-damaged buildings including metabolic products of toxigenic fungi and mold-derived inflammatory agents can lead to a deficiency or imbalance of many hormones, such as active T3 hormone. Since the 1970s, the synthetic prohormone, levothyroxine (LT4), has been the most commonly prescribed thyroid hormone in replacement monotherapy. It has been presumed that the peripheral conversion of T4 (3,5,3′,5′-tetraiodothyronine) into T3 (3,5,3′-triiodothyronine) is sufficient to satisfy the overall tissue requirements. However, evidence is presented that this not the case for all patients, especially those exposed to indoor air molds. This retrospective study describes the successful treatment of nine patients in whom NTIS was treated with T3-based thyroid hormone. The treatment was based on careful interview, clinical monitoring, and laboratory analysis of serum free T3 (FT3), reverse T3 (rT3) and thyroid-stimulating hormone, free T4, cortisol, and dehydroepiandrosterone (DHEA) values. The ratio of FT3/rT3 was calculated. In addition, some patients received adrenal support with hydrocortisone and DHEA. All patients received nutritional supplementation and dietary instructions. During the therapy, all nine patients reported improvements in all of the symptom groups. Those who had residual symptoms during T3-based therapy remained exposed to indoor air molds in their work places. Four patients were unable to work and had been on disability leave for a long time during LT4 monotherapy. However, during the T3-based and supportive therapy, all patients returned to work in so-called “healthy” buildings. The importance of avoiding mycotoxin exposure *via* the diet is underlined as DIO2 genetic polymorphism and dysfunction of DIO2 play an important role in the development of symptoms that can be treated successfully with T3 therapy.

## Introduction

Long-term exposure to molds in water-damaged buildings (WDB) has been associated with numerous health problems including allergic airway symptoms (1–3), fungal sinusitis (1, 2, 4), abnormalities in T and B cells (5–7), infection sensitivity (6, 8), asthma (9–11), respiratory infections (3, 11, 12), central and peripheral neuropathy and polyendocrinopathy (8), neurologic symptoms (1, 4, 13), neuropsychological cognitive dysfunction (CD) (14–16), neuropsychiatric symptoms (3, 14, 17), and chronic fatigue (CF) (6, 14, 18). It is now well established that mold and mycotoxins are important constituents of the milieu in WDB and that they can provoke a huge spectrum of illnesses (3, 8, 12, 15, 18–37).

Exposure to volatile organic compounds including metabolites produced by toxigenic fungi, some of which are inflammatory agents, can lead to a deficiency or imbalance of many hormones, such as insufficient amounts of the active form of thyroid hormone, commonly abbreviated to T3 hormone (38, 39).

Thyroid hormones play a very important role in development, growth, and glucose–fat–protein metabolic homeostasis in all tissues by affecting the expression of many genes. It has been shown that the most important factors in thyroid hormone regulation are the activities of the three deiodinase enzymes (DIO 1, 2, 3). In particular, DIO2 regulates the activities of thyroid hormone action by metabolizing the precursor molecule thyroxine (T4) that is secreted by the thyroid gland into the biologically active molecule, T3. Two of the deiodinases (DIO1, DIO2) contain selenium and are responsible for transforming T4 either into its active metabolite, i.e., T3 or to an inhibitory reversed T3 form, rT3 (DIO3) (40–43). The importance of DIO enzymes in thyroid hormone homeostasis has become increasingly clear by experiments conducted in DIO knockout mice (44). DIO enzymes affect the thyroid hormone regulation by controlling thyroid hormone homeostasis at the cellular level, such as in the case of symptoms in mold exposure or in other situations in which there is a lack of active T3 hormone in the peripheral tissues or brain (45). A deficiency of active cellular T3 hormone has been described as a non-thyroidal illness syndrome (NTIS) (46). The patients with this disease presents with normal function of thyroid or with required exogenous T4 with normal thyroid-stimulating hormone (TSH), free T4 (FT4), and free T3 (FT3) values in the blood, but still with symptoms of hypothyroidism. Importantly, the major part of T3 is generated locally from T4 by DIO2 in most tissues of the body and in the brain, especially at the hypothalamus–pituitary level (47).

The toxins released by the microbes living in damp buildings can induce oxidative stress (OS). OS has been proposed to be one of the most important mechanisms behind the adverse health outcomes associated with living in a damp indoor environment. One of the putative consequences of mycotoxin-induced OS is a blockade of crucial mitochondrial functions (47). OS may cause cytotoxic, genotoxic, and inflammatory responses by increasing the production of reactive oxygen species (18, 48).

Oxidative stress also impacts negatively on various hormonal influences, e.g., causing antioxidant imbalance and impairing the functions of the deiodinase enzymes. For example, OS reduces the capacity of DIO2 to convert thyroxine (T4) into its biologically active form of T3. Different defense mechanisms that protect against the free radical damage have been characterized in various cellular localizations, including the endoplasmic reticulum, mitochondria, plasma membrane, peroxisomes, and cytosol.

There are several enzymes such as superoxide dismutase, catalase and glutathione peroxidase, and transition-metal binding proteins, which rapidly inactivate free radicals (49).

Thus, OS can be defined as a failure of the antioxidant system to cope with the excess of free radicals. One putative hypothesis is that OS facilitates the development of hypothyroidism or rather a lack of availability of the T3 hormone at the tissue level, the so-called NTIS (38). In the 1970s, much of the basic biochemistry of thyroid metabolism was clarified (46, 50). It has been postulated that the level of rT3 and the FT3/rT3 ratio correlate with tissue DIO activities and reflect the peripheral metabolism of thyroid hormones. In a normal physiological situation in the human body, the amount of FT3 should be about 2–2.5 times higher than that of rT3; this represents the optimal level of active T3 in peripheral and brain tissues. In the normal physiological situation, rT3 is metabolized 2.5 times more rapidly than T3, and therefore, the FT3/rT3 calculated ratio should be at least around 2–2.5 (50) (or 20–25, depending on the form or expression).

In insulin-resistant patients, the T3/rT3 ratio is significantly increased in comparison to the corresponding value in insulin-sensitive controls (51). In the treatment of obesity, it has been suggested that attention should be paid to correcting the uncoupling of the mitochondrial respiratory chain (52). For example, in an experimental diet-induced obesity model, higher rT3 concentrations were detected in obese animals, one consequence of which might be reduced by oxygen consumption (53).

Patients who have been exposed for a prolonged time to indoor air molds have high serum levels of rT3 (unpublished observation). This indicates an imbalance between rT3 and FT3 and decreased tissue metabolism of T4 to be converted to T3, in other words NTIS. In these patients, DIO2 does not function properly (45), therefore T3 therapy is indicated. The rationale for T3 therapy is: T3 is biologically active hormone that does not require activity of the DIO2 which is needed for conversion from endogenous prohormone T4 to active T3 hormone, or when exogenous levothyroxine (LT4) monotherapy is administered. I will describe the treatment of patients with diagnosed NTIS due to long-term exposure to dampness microbiota.

## Materials and Methods

### Patients and Treatment

Nine female patients, aged 31–49 years, with a history of mold exposure and a variety of symptoms compatible with NTIS were enrolled into this retrospective study. The patients received LT4 monotherapy without success. Data have been collected since 2012 in a private medical clinic. Informed written consent was obtained from all of the patients.

**Figure 1:**
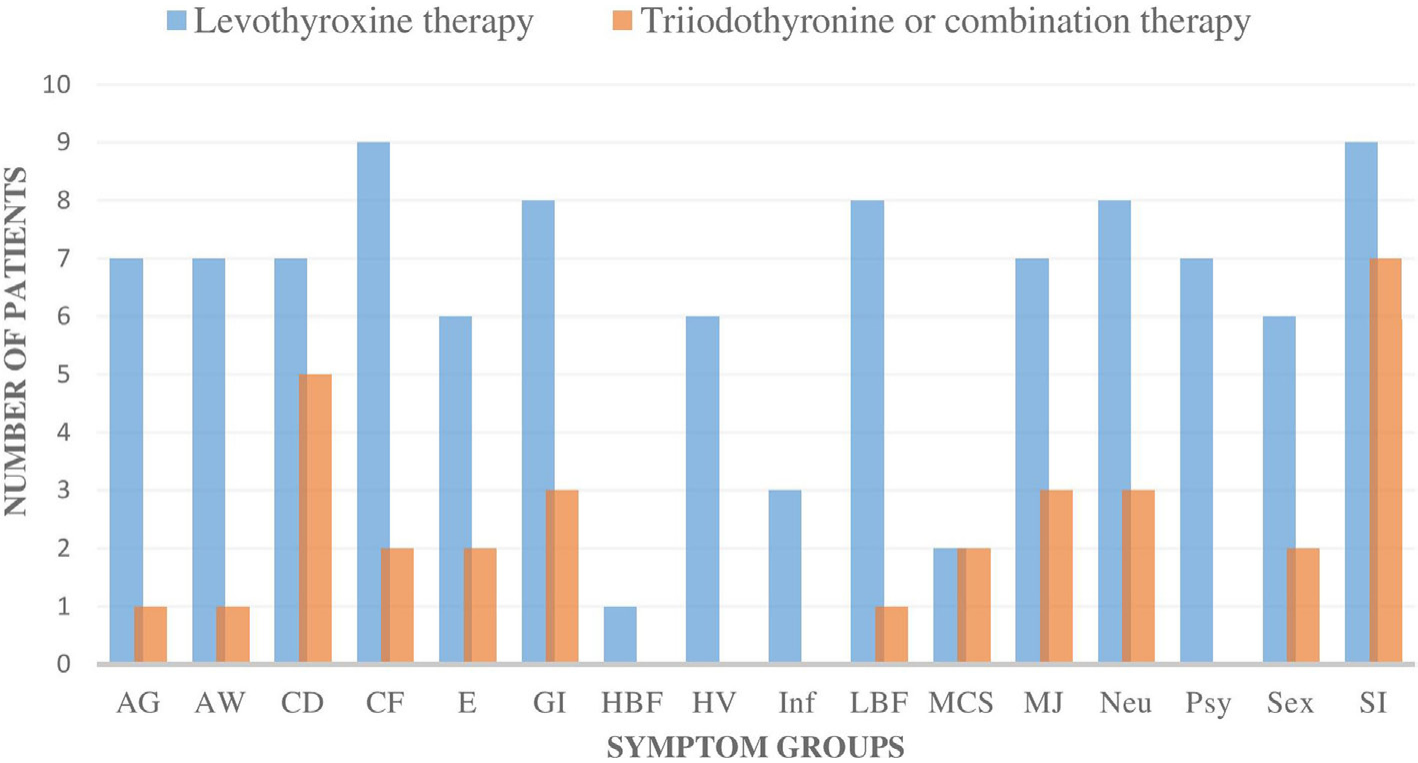
The number of all the individual symptom groups during levothyroxine monotherapy and during triiodothyronine monotherapy (T3) or combination therapy (T3 + T4) in all nine patients. All of the nine patients had fewer symptoms in all of the symptom groups during the T3-based therapy. In addition, all of the symptoms became either milder or were totally eliminated during T3-based therapy. Symptoms were categorized as follows: allergic symptoms (AG), airway symptoms (AW), cognitive dysfunction (CD), chronic fatigue (CF), edema/swelling (E), gastrointestinal symptoms (GI), high body temperature/feeling cold and feverish (HBF), heart/vascular disorder (HV), infection sensitivity (Inf), low body temperature/feeling cold, freezing (LBF), multiple chemical sensitivity (MCS), muscle and joint symptoms (MJ), neurological symptoms (Neu), psychological symptoms (Psy), imbalance in sex hormones (Sex), stress intolerance (SI: physical, psychological, or social stress).

The patients’ symptoms were categorized as follows: allergic symptoms (AG), airway symptoms (AW), CD, CF, edema/swelling (E), gastrointestinal symptoms (GI), high body temperature/feeling cold and feverish (HBF), heart/vascular disorder (HV), infection sensitivity (Inf), low body temperature/feeling cold, freezing (LBF), multiple chemical sensitivity, muscle and joint symptoms (MJ), neurological symptoms (Neu), psychological symptoms (Psy), imbalance in sex hormones (Sex), stress intolerance (SI: physical, psychological, or social stress) (Figure 1). Evaluation of symptoms was done on the basis of careful interview and clinical status.

Patients were instructed about aspects of nutritional therapy: adherence to a gluten-free diet, regular use of nutritional supplements, low-dose hydrocortisone, and/or dehydroepiandrosterone (DHEA) when required, and adrenal supportive therapy for a few months. If the above described treatment was not effective enough, T3-based therapy was initiated.

### Laboratory Analysis

Serum TSH, FT4, FT3, TPOAb, TyglAb, cortisol, and DHEA were assayed by Genova Diagnostics (Asheville, NC, USA) and the United Medix Laboratories Ltd. (Helsinki, Finland). The level of reverse T3 was assayed by Mayo Clinic (Scottsdale, AZ, USA). DIO2 was measured in the ZRT Laboratory (Beaverton, OR, USA) and in the United Medix Laboratories Ltd. (Helsinki, Finland). Saliva concentrations of cortisol and DHEA were assayed by Genova Diagnostics (Asheville, NC, USA) and ZRT Laboratory (Beaverton, OR, USA). The FT3/rT3 ratio was calculated according to the formula (FT3 × 6.51/rT3), where FT3 is picomoles per litre and rT3 is nanograms per deciliter.

### Mold Exposure

All patients reported an exposure to indoor air molds. The duration of exposure to molds ranged from 5 to 27 years (average 11.4 years). It is worth stressing that all of the patients reported that their disease started during a long exposure to a moldy environment and that their health condition deteriorated significantly during periods of re-exposure. In all cases, there was a correlation of symptoms with the growth of dampness microbiota in the buildings as well as visible evidence of water damage. Mold growth and indoor air investigations were done by accredited laboratories using accepted culture techniques with conventional isolation media and quantitation of colony forming units per, e.g., cubic meter (54). The majority of the damaged buildings were dismantled or renovated afterward.

## Results

Seven patients had been already diagnosed by hypothyreosis with clear or borderline levels of TSH and FT4; two patients had normal TSH and FT4 concentrations before initiating LT4 monotherapy. Four patients were not able to increase the prescribed dose of LT4 to the required level because of side effects (Table 3). Two patients had Hashimoto’s thyroiditis, one patient had goiter, and one had been semi-thyroidectomized because of goiter. Two patients had been diagnosed with newly onset asthma during the time when they were exposed to indoor air molds. Three of the nine patients had a deficiency or borderline levels of cortisol. Two patients had low or borderline cortisol and DHEA levels. Only three patients had normal cortisol and DHEA levels in serum, although they had symptoms consistent with an adrenal insufficiency (SI) (Table 3). While on LT4 monotherapy, six patients presented with very severe/severe imbalance in the FT3 and rT3 levels with FT3/rT3 ratios ranging from 0.75 to 1.3. Two patients had a moderate imbalance (range 1.43–1.61) and only one patient had a normal ratio (2.5) while consuming a low dose (50 µg) of LT4 monotherapy (Table 1).

**Table 1:** Details during levothyroxine (LT4) monotherapy.

Patient	LT4 dose (μg)	LT4 therapy problems	Thyroid-stimulating hormone (TSH) (mU/l)	Free T4 (FT4) (pmol/l)	Free T3 (FT3) (pmol/l)	rT3 (ng/dl)	FT3/rT3
1	62.5	If dose elevated: side effects	2.5	17	4.9	26	1.23
2	50		2	18	6	46	0.85
3	200		0	28	6.8	59	0.75
4	75	If dose elevated: side effects	2.5	17	5.2	29.5	1.15
5	200		0	16	4.2	21	1.3
6	100	If dose elevated: side effects	0	12	3.5	22.3	1.02
7	50		2	14	4.6	12	2.5
8	100		1.3	16	4.6	21	1.43
9	100	If dose elevated: side effects	6.11	15	4.2	17	1.61

*Doses of levothyroxine monotherapy (T4) and values of TSH, FT4, FT3, rT3, and calculated FT3/rT3 ratio (normal ≥ 2–2.5) before initiating T3 monotherapy or combination therapy (T3 + T4)*.

**Figure 2:**
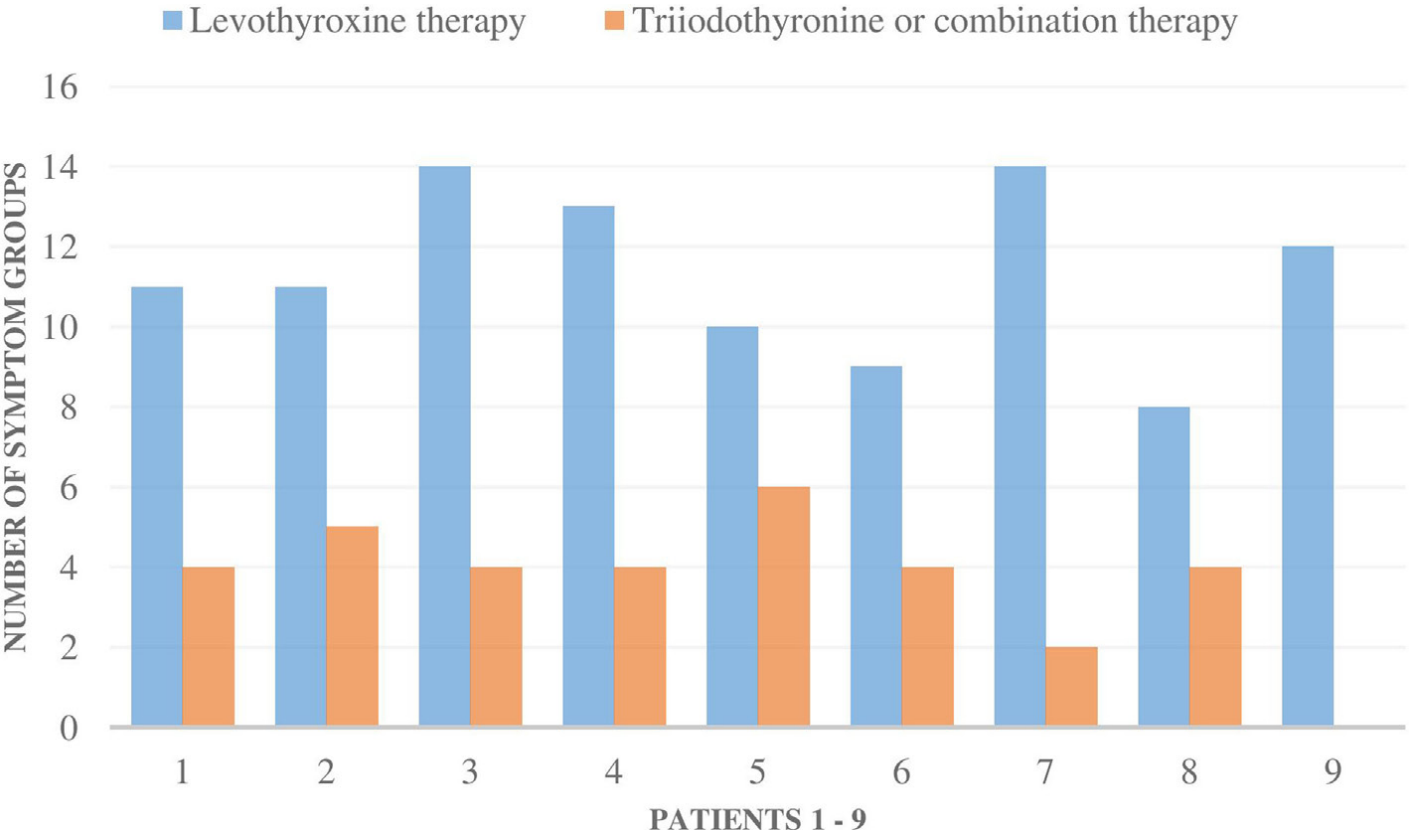
The number of all 16 symptom groups [allergic symptoms (AG) to stress intolerance] in each of the nine patients during levothyroxine monotherapy and during triiodothyronine monotherapy (T3) or combination therapy (T3 + T4). Special remarks: patient 1–5, 8: all the symptoms were milder during T3-based therapy; patient 1: still a mild mold exposure at work, patient 2: strong symptoms if mold exposure, patient 3: need less asthma medication, no airway symptoms (AW) during T3 therapy, patient 4: strong mold exposure period just recently, gets AW and AG symptoms immediately if mold exposure, patient 9: if not mold exposure, all the symptoms stay away.

The symptoms of all nine patients were relieved in all of the symptom groups when they were administered T3-based therapy (Figures 1 and 2). Those patients, who had residual symptoms during T3-based therapy, were continually exposed to molds at their work places (Figure 2). Four patients were unable to work and had been on a disability leave during LT4 monotherapy. However, during the T3-based and supportive therapy, all of these patients returned to work in so-called “healthy” buildings and all of these patients returned to work in so-called “healthy” buildings and were able to tolerate small amounts of mold and toxins.

The transition from LT4 monotherapy to T3-based therapy was conducted over a 2-week period when the patient’s symptoms were monitored to adjust to an individually appropriate T3 dosage. T3 monotherapy was prescribed to four patients in a dosage range from 30 to 55 μg/day, with these doses subdivided to be taken three times in a day. Combination therapy with synthetic T3 and LT4 thyroid hormones (T3 12.5–35 µg and LT4 50–75 µg) was prescribed to four patients and one patient received biological thyroid hormone extract (LT4 + T3). Satisfactory results were achieved within 3–36 months (average 10.1), but a complete curative response was documented in six patients within a shorter time, between 3 and 6 months (Table 2). The initiation of T3-based therapy relieved all of the symptoms in all of the nine patients. T3-based therapy lessened SI symptoms to a variable degree in all nine patients; they tolerated stress much better especially with the adrenal support (55–58). It is noteworthy that those patients who presented with HV, psychological (Psy), or infection sensitivity (Inf) symptoms during LT4 monotherapy became asymptomatic during T3-based therapy (Figures 1 and 2). Body temperature normalized and patients who presented with allergic or airway symptoms became asymptomatic in the majority of cases. Those patients (3/9) who ignored adherence to a strict naturally gluten-free diet continued to have GI symptoms despite of thyroid hormone correction. Patients with SI may have a comorbidity with latent adrenal insufficiency and they benefited from adrenal support provided as supplementation with physiological dosages of hydrocortisone and/or DHEA (Table 3).

**Table 2:** Details during T3 monotherapy or combination therapy.

Patient	T3 or T3 + T4 dose (μg)	Thyroid-stimulating hormone (TSH) (mU/l)	Free T4 (FT4) (pmol/l)	Free T3 (FT3) (pmol/l)	Duration of therapy/months
1	T3 30	1.6	6.1	4	6
2	T3 30	0	4.5	5.1	4
3	T3 55	0	<3	5.1	4
4	T3 30 + T4 115	0	12	5.1	14
5	T3 20 + T4 75	3.3	9.3	3.2	6
6	T3 50	0	<3	5.1	36
7	T3 30 + T4 50	2.2	8.1	3.6	6
8	T3 30 + T4 50	0.06	6.2	4.6	8
9	T3 12.5 + T4 62	1.43	10.42	3.9	3

*Doses of triiodothyronine monotherapy (T3) or combination therapy (T3 + T4) and control values of TSH, FT4, and FT3 in symptom-guided thyroid hormone therapy when the successful results were achieved (duration of the therapy in months)*.

**Table 3:** Details of the medical records before T3 or combination therapy.

Patient	Mold exp/years	Hashimoto	Goiter	Asthma	Low cortisol	Low dehydroepiandrosterone (DHEA)	DIO2	Glucocort. (mg/day)	DHEA (mg/day)
1	10	No	No	No	No	No	CT	No	No
2	27	No	No	No	Yes	No	nd	HC 25	No
3	7	Yes	No	Yes	Yes	No	nd	HC 30	No
4	10	No	Yes	No	Yes	Yes	nd	HC 30	25
5	5	No	No	No	No	No	TT	No	No
6	12	Yes	No	No	Yes	Yes	nd	HC 25 + Pred 5	50
7	6	No	Yes	No	Yes	No	TT	HC 25	No
8	5	No	No	No	No	No	CC	No	No
9	25	No	No	Yes	No	No	nd	No	No

*Details of mold exposure time in years, associate diseases, DIO2 polymorphism (TT is genetically normal, individuals with either CT or CC have impaired function of DIO2 enzyme), cortisol/DHEA levels (from serum and/or saliva), and substitution of cortisol. If the levels of cortisol and DHEA were low, substitution with hydrocortisone, prednisolone, and supplementation with DHEA*.

## Discussion

At present, there is no unanimous consensus on diagnostic criteria or efficacious treatment modalities for mold-exposed individuals. It has been estimated that as many as 14.5% of residents in Finland suffer from sick building syndrome (59). The Finnish authorities are reluctant to clarify the reasons for this problem. Instead, they claim that the patients’ symptoms are medically unrelated, unexplained, or possibly psychiatric, with one exception which has accepted the possibility that a moldy environment may evoke airway symptoms (60).

Hypothyroidism and NTIS appear to be common among patients with dampness and mold hypersensitivity syndrome (DMHS) (unpublished observation); this is often disregarded and neglected. It is well known that hypothyroidism is more common in females. In this study, all patients were females. The reasons may be that more women than men work in public institutions, which are often infested with indoor air molds. I have tackled this problem by treating patients with active T3 hormone, as well as supporting adrenal gland function and providing nutritional advice.

In an animal model of primary hypothyroidism, it has been demonstrated that the LT4 monotherapy could not achieve systemic euthyroidism (44). Thyroidectomized rodents treated with LT4 at doses that normalized serum TSH concentrations exhibited relatively lower serum T3 and higher serum T4 levels. Furthermore, markers of hypothyroidism could be identified in their brains, skeletal muscles, and livers. Measurements of thyroid hormone levels in serum do not correlate with the hormone levels in tissues; this is the case not only in animal studies *in vivo* but also in human studies *in vitro* (44). In humans, it has been shown that NTIS, the level of rT3, and the T3/rT3 ratio were correlated with postmortem tissue DIO enzyme activity (58). It is of utmost importance to understand the crucial role of DIO2, as well as measuring FT3/rT3 ratio, which are useful laboratory markers.

Successful treatment of DMHS patients required stringent adherence to a gluten-free diet. They were allowed to eat only naturally gluten-free cereals, but not gluten-stripped cereals marketed as “gluten-free.” The rationale for this approach is that wheat, rye, spelt, and barley can contain various toxic compounds, such as trichothecene mycotoxins (61) and ergot alkaloids (62–64), as well as metabolites from *Aspergillus* and *Penicillium* and other fungi which exist in cereals (65). The ergot alkaloid producing fungi, *Claviceps purpurea*, often live in symbiosis with gluten-containing cereal grains and *Penicillium* and *Aspergillus* strains have been demonstrated to produce ergot alkaloids in broth culture (66). It has been claimed that simultaneous inhalation and oral digestion of these toxins seem to potentiate the mold-induced symptoms. Furthermore, possibly gastric enzyme activity is impaired by the amylase and trypsin inhibitors (ATI) present in gluten cereals (67). These ATI structures in grains hinder digestion in the gastrointestinal tract. Gluten-containing cereals make up a large part of the everyday diet of most Europeans. It has been postulated that even a low toxin load is harmful; chronic exposure to mycotoxins has been claimed to be a crucial factor in causing a dysfunction of mitochondrial energy production (68). The importance of T3 for stimulating mitochondrial activity was presented by Forini et al. in their animal model, T3 decreased the size of cardiovascular infarction and prevented heart failure (69).

Another important mechanism in NTIS is DIO2 polymorphism. DIO2 is present in all cells of the human body. Patients who are heterozygotes (CT) or homozygotes (CC) have defective DIO2 activity, and they will benefit from T3-based therapy compared to LT4 monotherapy (70). Presumably an improved DIO2 enzyme activity explains why part of the patients recovered on nutritional supplementation especially with selenium. T3-based therapy is indicated in situations when the FT3/rT3 ratio is abnormal due to overproduction of rT3 and when the patient presents with symptoms compatible with hypothyroidism or NTIS (71). According to U.S. Food and Drug Administration, all thyroid hormone drugs, including T3 monotherapy are safe, and can be utilized as replacement or supplemental therapy in patients of any age, and also during pregnancy (72–79).

## Conclusion

This article describes the successful treatment of patients with hypothyroidism and NTIS. The disease developed after prolonged and cumulative or massive exposure to indoor air dampness microbiota. The treatment was based on full laboratory assessment of thyroid and adrenal hormones as well as careful interviewing and clinical monitoring. DMHS with NTIS is a devastating condition caused by OS and toxicosis, which should be recognized and treated appropriately.

## Ethics Statement

Ethical clearance was not needed because this study was a retrospective study using the medical records. Samples were taken during treatment and the patient provided written informed consent and provided permission to use their data for scientific purposes.

## Author Contributions

TS has interviewed, treated, and evaluated all the patients and wrote the study.

## Conflict of Interest Statement

TS is one of the owners of a private clinic in Tampere, Finland.
